# Disentangling the varying associations between systolic blood pressure and health outcomes in the very old: an individual patient data meta-analysis

**DOI:** 10.1097/HJH.0000000000003219

**Published:** 2022-07-11

**Authors:** Jonathan M.K. Bogaerts, Rosalinde K.E. Poortvliet, Veerle M.G.T.H. van der Klei, Wilco P. Achterberg, Jeanet W. Blom, Ruth Teh, Marama Muru-Lanning, Ngaire Kerse, Anna Rolleston, Carol Jagger, Andrew Kingston, Louise Robinson, Yasumichi Arai, Ryo Shikimoto, Jacobijn Gussekloo

**Affiliations:** a Department of Public Health and Primary Care, Leiden University Medical Center; b Department of Internal Medicine, Section Gerontology and Geriatrics, Leiden University Medical Center, Leiden, The Netherlands; c School of Population Health; d James Henare Maori Research Centre, University of Auckland, Auckland; e The Centre for Health, Tauranga, New Zealand; f Population Health Sciences Institute, Newcastle University, Newcastle upon Tyne, UK; g Center for Supercentenarian Medical Research, Keio University School of Medicine, Tokyo, Japan

**Keywords:** activities of daily living, aged, antihypertensive, blood pressure, body mass index, cardiovascular, cognition, frailty, grip strength, older adults

## Abstract

While randomized controlled trials have proven the benefits of blood pressure (BP) lowering in participating octogenarians, population-based observational studies suggest an association between low systolic blood pressure (SBP) and faster overall decline. This study investigates the effects of BP-lowering treatment, a history of cardiovascular diseases (CVD), and cognitive and physical fitness on the associations between SBP and health outcomes in the very old.

**Methods:**

Five cohorts from the Towards Understanding Longitudinal International older People Studies (TULIPS) consortium were included in a two-step individual participant data meta-analysis (IPDMA). We pooled hazard ratios (HR) from Cox proportional-hazards models for 5-year mortality and estimates of linear mixed models for change in cognitive and functional decline. Models were stratified by BP-lowering treatment, history of CVD, Mini-Mental State Examination scores, grip strength (GS) and body mass index (BMI).

**Results:**

Of all 2480 participants (59.9% females, median 85 years), median baseline SBP was 149 mmHg, 64.3% used BP-lowering drugs and 47.3% had a history of CVD. Overall, higher SBP was associated with lower all-cause mortality (pooled HR 0.91 [95% confidence interval 0.88–0.95] per 10 mmHg). Associations remained irrespective of BP-lowering treatment, history of CVD and BMI, but were absent in octogenarians with above-median MMSE and GS. In pooled cohorts, SBP was not associated with cognitive and functional decline.

**Conclusion:**

While in the very old with low cognitive or physical fitness a higher SBP was associated with a lower all-cause mortality, this association was not evident in fit octogenarians. SBP was not consistently associated with cognitive and functional decline.

## INTRODUCTION

Multiple large-scale randomized controlled trials (RCT) and meta-analyses of these trials underline the crucial role of antihypertensive treatment in primary and secondary prevention of cardiovascular diseases (CVD) [1,2]. This increasing amount of evidence permits an optimal risk-based approach up to approximately the age of 75–80 years old. While certain trials do include octogenarians [3–5], the very old with multimorbidity are largely underrepresented in the published interventional studies on hypertension management [6–8]. The selection of more healthy participants limits the application of RCT evidence on all individuals of this highly heterogenic population. Community-based longitudinal studies with an observational design reflect a more representative sample of the aged population. Several of these observational studies [9–12], however, question the beneficial effects of intensive lowering of systolic blood pressure (SBP) on outcomes such as survival and cognition in the very old, possibly attributed to physiological changes in advanced age.

Recent guidelines acknowledge the paucity of evidence and clearly reflect the discrepancies between the included populations and thereby apparently conflicting implications of both observational and interventional studies. While certain guidelines recommend one general treatment goal for those above and below the age of 80 years [13,14], others de-intensify treatment in octogenarians [15] or even endorse a lower target for adults above the age of 75 years [16,17]. While the current guidelines do not succeed in giving a uniform advice based on age alone, they agree that, with increasing frailty or vulnerability, targets should be adapted on base of clinical judgment [13–15,18].

With a scientific debate concerning intensive antihypertensive treatment when cognitive and physical function decline and clinical guidelines emphasizing the difficulties of geriatric hypertension management [19,20], the question remains how in different subgroups of octogenarians SBP and overall health outcomes are related. Therefore, we aim to investigate whether individual variation characterized by BP-lowering treatment, history of CVD, and cognitive and physical fitness affect the associations between SBP and health outcomes (mortality, cognition and functional status) in octogenarians. In this meta-analysis, we combine individual participant data from the Towards Understanding Longitudinal International older People Studies (TULIPS) consortium, a unique collaboration between four large, international prospective studies of octogenarians.

## METHODS

### Design and setting

The data for this individual participant data meta-analysis (IPDMA) was provided by five cohorts from four studies of the TULIPS consortium: the Leiden 85-plus study, the Life and Living in advanced Age: a Cohort Study in New Zealand (LiLACS NZ) study, providing a Maori and non-Maori cohort, the Newcastle 85+ study, and the Tokyo Oldest Old Survey on Total Health (TOOTH). Appropriate ethical approval from respective authorities was obtained for all studies. Details of individual study variables used for the present analysis are summarized in Supplementary Table 1 (see Supplemental Digital Content 1).

#### The Leiden 85-plus study

Between September 1997 and September 1999, all inhabitants of Leiden (the Netherlands) reaching the age of 85 (*n* = 705) were approached to participate in a 5-year follow-up study with yearly visits including interviews and performance of functional tests [21]. Baseline data for this IPDMA were available for 557 of the 599 participants.

#### Life and Living in advanced Age: a Cohort Study in New Zealand (LiLACS NZ) study

The LiLACS NZ study is composed of two distinct parallel cohorts, one with solely Maori participants (defined by whether identifying themselves as Maori), the indigenous people of New Zealand, and one with only non-Maori participants. An equal sample of Maori people was purposefully recruited to enable equal explanatory power between the two cohorts [22]. In 2010, all Maori aged 80–90 years of age and all non-Maori aged 85 years living in the Lakes or Bay of Plenty District Health Board area (*n* = 1636) were eligible [23]. In total, 421 Maori and 516 non-Maori, of the 1636 potential participants, were enrolled in this cohort study with annual structured questionnaires and physical health assessments. For the LiLACS NZ study five waves after baseline were used. Baseline data for this IPDMA were available for 226 Maori and 356 non-Maori participants.

#### Newcastle 85+ study

In 2006, 1470 citizens born in 1921, who were registered with a participating general practitioner (GP) in Newcastle/North Tyneside area (the United Kingdom), were invited to undergo a battery of questionnaires, measurements, and function tests at baseline, 18 months (apart from cognitive function), 3 and 5 years of follow-up [24]. Baseline data for this IPDMA were available for 834 of the 852 participants.

#### Tokyo Oldest Old Survey on Total Health

Between March 2008 and November 2009, study recruitment took place in the wards of Shinjuku and Minato, and the east half of the Shibuya in the Tokyo Metropolitan area (Japan). A random selection of 3320 residents aged 85 years and older resulted in a recruitment of 542 participants with both an interview and a medical examination at baseline [25]. TOOTH provided follow-up data on 3 and 6 years after baseline. Baseline data for this IPDMA were available for 536 of the 542 participants.

### Participants

Four prespecified exclusion criteria were applied. First, we excluded all participants who were missing one of the following cardiovascular baseline data: SBP, BP-lowering drugs and history of CVD (*n* = 421). Finally, to avoid effect overestimation due to the association between terminal illness and progressively declining blood pressure, we excluded participants who died in the first 90 days after baseline visit (*n* = 29).

### Data collected at baseline

#### Socio-demographic characteristics

In all cohorts, baseline data per participant on age, sex, diabetes mellitus (DM) and current tobacco use (yes/no) were collected.

#### Blood pressure

In all cohorts, both systolic and diastolic blood pressure (DBP) at baseline were collected multiple times, resulting in one average SBP and DBP variable from the available reading(s) of the individual participant.

#### Blood pressure lowering drugs

In all cohorts, the use of BP-lowering drugs was recoded (yes/no) into prescription of one or more drugs of four subgroups from the Anatomical Therapeutic Chemical (ATC) Classification System of the World Health Organization [26]. The subgroups were: diuretics (ATC C03), beta blockers (ATC C07), calcium channel blockers (ATC C08), and agents acting on the renin-angiotensin system (ATC C09). In the Leiden 85-plus study and the LiLACS NZ study, the use of antihypertensives (ATC C02; the subgroup containing, for example centrally acting antiadrenergic agents), was additionally included. In TOOTH, the use of alpha blockers was added to the BP-lowering variable.

#### History of cardiovascular diseases

In all cohorts, a history of CVD at baseline (yes/no) included stroke, transient ischemic attack, myocardial infarction and angina pectoris. Excluding TOOTH, all other studies additionally collected data on the presence of peripheral artery disease and any surgery related to coronary or peripheral artery disease.

#### Cognitive fitness

In all cohorts, the Mini-Mental State Examination (MMSE) at baseline was used as a proxy for cognitive fitness [27]. The MMSE combines 19 items to evaluate cognitive function, with a maximum summed score of 30. Lower scores correlate with worse cognition. The MMSE score was defined high when the participant scored above the median of the individual cohort. In the Leiden 85-plus study the median MMSE score was 26 points. In the LiLACS NZ study (both Maori and non-Maori) and the Newcastle 85+ study, the median score was 28 points. In TOOTH, the median score was 27 points. When the baseline MMSE score was missing, it was re-classified as low to enable inclusion.

#### Grip strength

In all cohorts, we operationalized physical fitness as grip strength (GS) with measurements in kilograms. GS was defined high when the participant scored above the sex-specific median of the individual cohort. In the Leiden 85-plus study, the sex-specific median GS was 20 kg and 30 kg for females and males, respectively. In the LiLACS NZ study (both Maori and non-Maori), the median GS was 19 kg and 31 kg. In the Newcastle 85+ study, the median GS was 15 kg and 28 kg. In TOOTH, the median GS was 16 kg and 25 kg. When GS measurements were missing, they were re-classified as low to enable inclusion.

#### Body mass index

Since a low body mass index (BMI) in advanced age is not only associated with declining physical [28], but also declining cognitive fitness [29], we added BMI as an additional operator of overall robustness in the very old. BMI was defined high when the participant scored above the median of the individual cohort. In the Leiden 85-plus study the median BMI was 26.8 kg/m^2^. In the LiLACS NZ study, the median BMI was 28.7 kg/m^2^ in the Maori and 26.4 kg/m^2^ in the non-Maori participants. In the Newcastle 85+ study, the median BMI was 24.2 kg/m^2^. In TOOTH, the median BMI was 21.4 kg/m^2^. When BMI was missing, it was re-classified as low to enable inclusion.

### Outcome variables

#### All-cause mortality

For the Leiden 85-plus Study, the LiLACS NZ study and the Newcastle 85+ study, date of death all originated from national registries [21,23,24]. For TOOTH, survival status was monitored through annual telephone contact. The time in days between baseline visit and date of death or censored at 1826 days (5 years) if death had not yet occurred, was used to calculate survival.

#### Cognitive function over time

In all cohorts, cognitive function was assessed over multiple waves with the MMSE. For the Leiden 85-plus Study and the LiLACS NZ study, five annual waves of data after baseline were available. For the Newcastle 85+ study and TOOTH, two waves of data after baseline were available (see Design and setting of cohorts).

#### Daily functioning over time

In the Leiden 85-plus study, activities of daily living (ADL) were measured with the Groningen Activity Restriction Scale (GARS) [30]. The LiLACS NZ study utilized the Nottingham Extended Activities of Daily Living (NEADL) questionnaire [31]. In the Newcastle 85+ study, ADL were evaluated with a sum score based on 17 activities. For TOOTH, the Lawton Instrumental Activities of Daily Living was utilized [32]. For the Leiden 85-plus study and the LiLACS NZ study, five annual waves of data after baseline were available. For the Newcastle 85+ study and TOOTH, respectively three and two waves of data after baseline were available (see Design and setting of cohorts).

We reversed the polarity of the scales that were used in the Leiden 85-plus study (GARS) and the Newcastle 85+ study (the 17 items-sum score), to standardize the direction of all scales (a higher score corresponds with better outcomes). Since each study used a different scale, we standardized by subtracting the baseline sample mean from each individual score and dividing that by the baseline standard deviation (SD) per cohort, resulting in a standardized *z*-score. In accordance with previous research, we defined a *z*-score of 0.5, which corresponds with an SD of 0.5 on the standardized score, as the minimal clinically important difference (MCID) [33].

### Statistical analyses

A two-stage IPDMA approach was employed for all analyses. In the first phase, analyses were performed at cohort level. In the second phase, the cohort level results were pooled for meta-analysis.

#### Cohort level analyses

Categorical variables were presented as frequency with a percentage of the total. For continuous variables, we used the median with interquartile range (IQR). We assessed associations between baseline SBP as continuous variable (exposure) and outcomes, being all-cause mortality over five years and change in rate of cognitive and functional decline over time. Analyses were corrected for sex (all cohorts) and age (only the Maori and TOOTH cohort).

The Cox proportional-hazards regression model was used for the five-year survival analysis and presented as a hazard ratio (HR) with 95% confidence intervals (CI) for every 10 mmHg increase of SBP. The proportionality of hazards assumption was visually checked after transformation of SBP to categories based on quartiles.

Repeated measures linear mixed-effect models with restricted maximum likelihood estimation and unstructured covariance matrix, were employed to assess the associations between SBP and both MMSE and ADL scores. To enable pooling of estimates across all cohorts, time was modeled in units of six months instead of years. The interaction term ‘SBP at baseline × time since baseline’, which represents the additional cognitive or functional change over time described per 10 mmHg increase of baseline SBP, with every 6 months since baseline, was the outcome. A random intercepts and random slopes model at the participant level was used.

After initial modeling, we stratified by the following binary baseline variables: BP-lowering treatment (yes/no), history of CVD (yes/no), MMSE baseline score (low/high), GS (low/high), and BMI (low/high) and repeated all analyses.

#### Pooled analyses

To respect the inter-cohort differences, we used a random-effects model with inverse-variance weighting for pooling of cohort effects. Statistical heterogeneity (*I*^2^) below 40% was defined as consistency between estimates [34]. When *I*^2^ was above 60%, we summarized the results in forest plots, but refrained from pooling. To assess the potential effect of the stratifications by BP-lowering treatment, history of CVD, baseline MMSE score, grip strength and BMI, we used the chi^2^-test for subgroup differences. A two-sided *P*-value of ≤ 0.05 was considered statistically significant.

#### Sensitivity analyses

We conducted two sensitivity analyses. Firstly, to assess the possible effect of reverse causality between SBP and mortality risk, we repeated the survival analysis with exclusion of all deaths within 1 year after baseline (additional deaths between 91 and 365 days after baseline).

Secondly, when a unidirectional association between the covariate ‘SBP at baseline’ and an outcome variable was absent, we repeated the initial analyses with SBP categories based on quartiles (lowest and highest quartile versus middle fifty group) in order to not miss a potential underlying J- or U-shaped association.

All analyses were performed at the Leiden University Medical Center, apart from the survival analysis of the Newcastle 85+ study, undertaken at Newcastle University with the same syntax. The cohort level analyses were performed using IBM SPSS Statistics version 25.0 (IBM, Armond, New York, USA). All pooled analyses were performed using Review Manager 5.3.5 (The Cochrane Collaboration, Copenhagen, Denmark).

## RESULTS

Table 1 summarizes the baseline characteristics for the individual cohorts and the combined study population of 2480 participants. The median participant age was 85 (IQR 85.1–85.9) years and 1486 (59.9%) participants were female. The median SBP in the combined study population was 149 (IQR 135–164) mmHg. Of all participants, 1594 (64.3%) were treated with BP-lowering drugs. A history of cardiovascular diseases was present in 1174 (47.3%) participants. The median MMSE baseline score was 27 (IQR 25–29; 24 missing) points. The median grip strength over all cohorts was 17 (IQR 14–20; 26 missing) for women and 28 (IQR 23–33; 7 missing) kg for men. The median BMI was 24.9 (IQR 21.9–28.2; 166 missing) kg/m^2^.

**TABLE 1:** Baseline characteristics of study participants arranged by cohort

	Leiden 85-plus	LiLACS NZ		Newcastle 85+	TOOTH	Combined
		Maori	Non-Maori			
Cohort	(*n* = 548)	(*n* = 225)	(*n* = 355)	(*n* = 824)	(*n* = 528)	(*n* = 2480)
Demographics						
Age in years, median (IQR)	85 (85.1–85.1)	82 (80.5–84.3)	85 (84.8–85.3)	85 (85.2–85.8)	87 (86.2–88.8)	85 (85.1–85.9)
Female, *n* (%)	358 (65.3)	133 (59.1)	183 (51.5)	513 (62.3)	299 (56.6)	1486 (59.9)
Diabetes mellitus, *n* (%)	86 (15.7)	64 (28.4)	55 (15.5)	116 (14.1)	98 (18.6)	419 (16.9)
Current smoker, *n* (%)	83 (15.1)	26 (11.6)	18 (5.1)	45 (5.5)	201 (38.1)	373 (15.0)
Cardiovascular characteristics						
SBP in mmHg, median (IQR)	154 (143–167)	142 (131–159)	149 (136–171)	150 (133–166)	143 (129–157)	149 (135–164)
DBP in mmHg, median (IQR)	77 (71–84)	79 (71–87)	81 (74–89)	74 (66–82)	77 (69–84)	77 (69–84)
Blood pressure lowering drugs, *n* (%)	301 (54.9)	175 (77.8)	249 (70.1)	567 (68.8)	302 (57.2)	1594 (64.3)
Any history of CVD, *n* (%)	252 (46.0)	143 (63.6)	216 (60.8)	449 (54.5)	114 (21.6)	1174 (47.3)
Cognitive function (Mini-Mental State Examination)						
Median (IQR)	26 (22–28)	28 (26–29)	28 (27–29)	28 (25–29)	27 (25–29)	27 (25–29)
Physical function						
Grip strength in kg, median (IQR) (female–male)						
Female	20 (15–22)	19 (17–24)	19 (15–22)	15 (12–19)	16 (14–19)	17 (14–20)
Male	30 (25–36)	31 (27–35)	31 (27–35)	28 (22–33)	25 (22–28)	28 (23–33)
Body mass index (BMI)						
Median (IQR) in kg/m^2^	26.8 (24.4–29.9)	28.7 (25.6–32.5)	26.4 (24.4–29.1)	24.2 (21.6–27.2)	21.4 (19.3–23.6)	24.9 (21.9–28.2)
Activities of daily living						
Questionnaire	GARS	NEADL	NEADL	SUM score	Lawton	Cohort-dependent
Median (IQR)	27 (21–39)	19 (16–20)	19 (17–20)	3 (1–7)	5 (4–5)	
*z*-score, median	-0.38	0.35	0.29	-0.37	0.55	0.37^‡^

CVD, cardiovascular diseases; DBP, diastolic blood pressure; GARS, Groningen Activity Restriction Scale; IQR, interquartile range; NEADL, Nottingham Extended Activities of Daily Living; SBP, systolic blood pressure.

‡ After reversal of the polarity of the scales that were used in the Leiden 85+ (GARS) and the Newcastle 85+ study (the 17 items-sum score). Missing data: diabetes mellitus (*n* = 105), current smoker (*n* = 33), MMSE (*n* = 24), grip strength (*n* = 33), body mass index (*n* = 166) and activities of daily living score (*n* = 18).

### All-cause mortality

**FIGURE 1:**
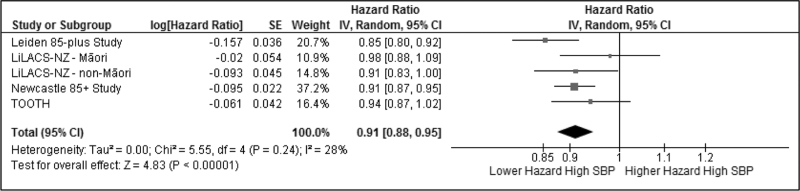
All-cause 5-year mortality with corrections for sex (all) and age (only the Maori and TOOTH cohort) calculated with Cox proportional-hazards regression models and presented as a hazard ratio with 95% confidence intervals for every increase of SBP with 10 mmHg. The hazard ratios of the individual cohorts were pooled using a random-effects models with inverse-variance weighting. CI, confidence interval; IV, inverse variance; SE, standard error.

During the 5 years follow-up, 972 (39.2%) participants died. The pooled and individual study HR with 95% CI from the initial model (only corrected for sex and age) are displayed in Fig. 1. Pooled over all cohorts, 5-year all-cause mortality was significantly lower with increasing SBP (HR 0.91 per 10 mmHg higher SBP, 95% CI 0.88–0.95). The HR ranged from 0.85 (95% CI 0.80–0.92) per 10 mmHg higher SBP in the Leiden 85-plus study to 0.98 (95% CI 0.88–1.09) in the LiLACS NZ Maori cohort.

The subgroup analyses for all-cause mortality (Table 2), when stratified by the prescription of BP-lowering drugs was too heterogenic to pool (*I*^2^ = 78%). The pooled HR for those not prescribed BP-lowering drugs was 0.88 per 10 mmHg higher SBP (95% CI 0.81–0.95). Stratification of the initial model by history of CVD did not change the association between increasing SBP and five-year all-cause mortality (*P*-value for difference = 0.37).

**TABLE 2:** Pooled stratified models of systolic blood pressure at baseline and all-cause 5-year mortality

	HR per 10 mmHg higher SBP	95% CI		*P* value of difference
Nonstratified				
Initial model	0.91	0.88	0.95	

Stratified by cardiovascular status				
BP-lowering drugs				
Yes	n/a: *I*^2^ = 78%			n/a
No	0.88∗	0.81	0.95	
History of CVD				
Yes	0.91	0.86	0.95	0.37
No	0.93	0.89	0.98	

Stratified by cognitive and physical fitness				
Baseline MMSE				
Low	0.90	0.87	0.93	0.02
High	0.97	0.92	1.03	
Grip strength				
Low	0.89	0.86	0.93	0.03
High	0.96	0.91	1.01	
Body mass index				
Low	0.90	0.87	0.94	0.55
High	0.92	0.87	0.98	

BP, blood pressure; CI, confidence interval; CVD, cardiovascular diseases; HR, hazard ratio; MMSE, Mini-Mental State Examination; n/a, not available. ∗: *I*^2^ = 40–60%. When not shown or not otherwise labeled: *I*^2^ <40%. Results from Cox proportional-hazards regression models presented as a hazard ratio with 95% confidence intervals for every increase of systolic blood pressure with 10 mmHg. Both initial and stratified analyses after pooling using random-effects models with inverse-variance weighting are presented, including subgroup differences per analysis. Models were corrected for sex (all) and age (only the Maori and TOOTH cohort).

In participants with a low MMSE score at baseline, a higher SBP was associated with a lower all-cause mortality (HR 0.90 [95% CI 0.87–0.93]), as opposed to those with a high MMSE score at baseline (HR 0.97 [95% CI 0.92–1.03]) with a *P*-value for the difference between both subgroups of 0.02. Likewise, in individuals with a low GS, a 10 mmHg higher SBP was associated with a decreasing all-cause mortality (HR 0.89 [95% CI 0.86–0.93]), while in participants with high GS this association was absent (HR 0.96 [95% CI 0.91–1.01]). The *P*-value for the difference between both subgroups was 0.03. BMI had no impact on the association between increasing SBP and 5-year all-cause mortality (*P*-value for difference = 0.55).

### Change of rates in cognitive and functional decline over time

**FIGURE 2:**
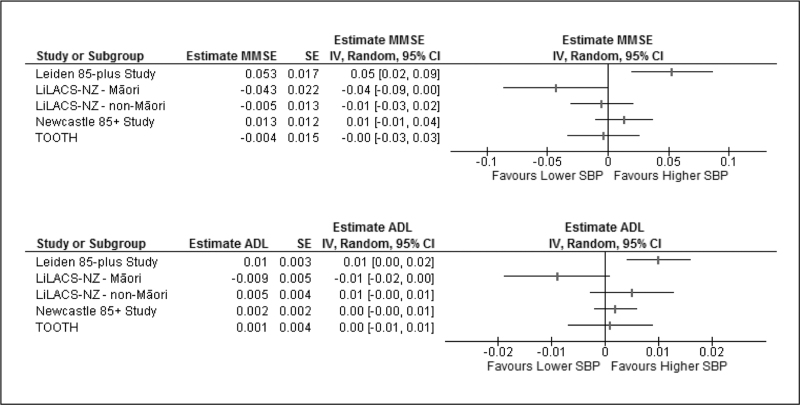
Repeated measures linear mixed model estimation of the change in Mini-Mental State Examination (MMSE) scores and standardized *z*-scores of activities of daily living (ADL) per increase of systolic blood pressure with 10 mmHg, with every six months since baseline. Models were corrected for sex (all) and age (only the Maori and TOOTH cohort). *I*^2^ MMSE = 72%. *I*^2^ ADL = 67%. CI, confidence interval; IV, inverse variance; SE, standard error.

The individual cohort estimates of the additional biannual change in MMSE and *z*-scores of ADL are displayed in Fig. 2. For cognition, the estimate ranged from –0.04 (95% CI –0.09–0.00) MMSE points per increase of SBP with 10 mmHg for the LiLACS NZ Maori cohort to 0.05 (95% CI 0.02–0.09) in the Leiden 85-plus study. For ADL, the estimate ranged from –0.01 (95% CI –0.02–0.00) *z*-scores per increase of SBP with 10 mmHg for the LiLACS NZ Maori cohort to 0.01 (95% CI 0.00–0.02) in the Leiden 85-plus study. Since there was high heterogeneity of both the estimate for MMSE (*I*^2^ = 72%) and the standardized ADL scores (*I*^2^ = 67%), we refrained from pooling.

High heterogeneity was also evident in the stratified analyses, except for the history of CVD and BMI (therefore the only fully pooled analyses). A history of CVD was not associated with a significantly faster or slower cognitive or functional decline with increasing SBP. Functional decline was significantly slower with every 10 mmHg increase of SBP (0.01 [95% CI 0.00–0.01] *z*-scores per 6 months since baseline) in octogenarians with a high BMI. Although statistically significant, a value of 0.01 corresponds with a 5-year difference in ADL of 0.1 SD of the standardized score, which is lower than the universally applicable MCID.

The estimates of all subgroup analyses are described in Supplementary Table 2, Supplemental Digital Content and Supplementary Table 3 (see Supplemental Digital Content 2 and 3).

### Sensitivity analyses

Exclusion of all deaths within 1 year after baseline did not considerably change the outcome of the original survival analysis (HR 0.92 [95% CI 0.89–0.95]), neither were the results of the stratified analyses substantially different.

Since a unidirectional association between SBP at baseline and change in rate of cognitive and functional decline was absent, we repeated the initial analyses with SBP categories based on quartiles. Cognitive decline (see Supplementary Figure 1A, Supplemental Digital Content 4) was not significantly faster in the lowest quartile (−0.03 [95% CI −0.10–0.05] MMSE points with every 6 months since baseline) in comparison with the middle 50% group. The difference between the highest SBP quartile and the middle 50% group was nonsignificant (−0.01 [95% CI −0.12–0.09] MMSE points with every 6 months since baseline).

For the lowest SBP quartile, functional decline (−0.02 [95% CI −0.03 to −0.01] *z*-score of ADL with every 6 months since baseline) was significantly faster in comparison with the middle 50% group (see Supplementary Figure 1B, Supplemental Digital Content 4). Although statistically significant, a value of −0.02 corresponds with a 5-year decline in ADL of 0.2 SD, being lower than the MCID. Therefore, we refrained from further subgroup analyses. The difference between the highest SBP quartile and the middle fifty percentage group was nonsignificant.

## DISCUSSION

In this IPDMA of five observational cohort studies including a total of 2480 octogenarians, SBP was inversely associated with all-cause mortality, independent of BP-lowering treatment, history of CVD and BMI. Individual variation in the very old described by MMSE scores or GS at baseline, in contrast, did modify this association. While in the very old with low cognitive or physical fitness a higher SBP was associated with a lower all-cause mortality, this association was not evident in fit octogenarians. No evidence of a unidirectional relationship between SBP and cognitive and functional decline over time was found. Overall, the high statistical heterogeneity of the associations between SBP and cognitive or functional decline over time, suggest underlying inter-cohort differences.

In accordance with RCT evidence concerning antihypertensive treatment in old age [4,5], our findings confirm that a higher SBP is not associated with a lower mortality in fit octogenarians, with variation between the populations. Interestingly, we found that certain markers of cognitive and physical fitness, as earlier reported in younger populations [35–38], affect the association between SBP and all-cause mortality in octogenarians. Contrary to earlier research [9,39], we found that in octogenarians without BP-lowering treatment, the association between low SBP and higher all-cause mortality remained significant. In contrast to the earlier reported relationship between lower SBP and accelerated decline in cognitive function and physical activities in the Leiden 85-plus Study [39,40], this IPDMA was not able to demonstrate a pooled association over all five cohorts. Moreover, for the LiLACS NZ Maori cohort, the opposite was shown, potentially suggesting that issues of equity for indigenous groups are important in advanced age [41]. The high heterogeneity of these outcomes found in old age (see Fig. 2), corresponding with the paradoxically positive, negative and lacking associations described in previous research [42,43], might indicate that, rather than SBP, other related factors are more directly associated underlying mechanisms [44]. Therefore, a one-size-fits-all recommendation may unfairly disadvantage some. Since a history of CVD and treatment with BP-lowering drugs did not modify the relation between SBP and all-cause mortality, not only cardiovascular risk factors but also cognitive and physical fitness need to be considered when managing hypertension in the very old.

In addition to RCTs, which have proven the positive effects of antihypertensive treatment on cardiovascular risk in old age, our IMPDA shows that, in robust octogenarians, low SBP is unrelated to higher all-cause mortality or faster cognitive and functional decline. Therefore, de-intensifying BP-lowering drugs solely on base of age, even in those aged 80 years and older, is not justifiable. Nevertheless, the net benefit of intensive BP-lowering treatment in frail older adults, who are at high risk for both cardiovascular events [45] and severe side-effects of hypotension [20], remains unclear [46]. Our finding that MMSE scores and GS modify the association between high SBP and all-cause mortality, underscore that management of hypertension in the very old should not be adapted on the base of chronological, but rather biological age. The question is whether in cognitively and physically less fit octogenarians maintaining a higher SBP, for example by withholding or withdrawing antihypertensive treatment, is beneficial, or that low SBP in these individuals is merely a marker of declining overall health.

This two-staged IPDMA of adults above the age of 80 years contributes to existing knowledge in several ways. The design of this meta-analysis combines data from five large community-based cohort studies with considerable representation of cultures and settings, resulting in a low risk of selection bias, but respecting the cohort-specific properties. The extensive measurements of variation between older participants permit investigation of the combined longitudinal association between SBP and multiple outcomes in population subgroups frequently excluded from current research.

The most important limitation of this study is its observational design, which does not allow causal interpretations of our findings. The pooled consistency, the parallels with previous research and the temporal relationship over 5 years that yielded after a sensitivity analysis, however, support the strength of the association found between high SBP and all-cause mortality, and its modification by MMSE and GS. Furthermore, we operationalized high cognitive and physical fitness as above median MMSE and GS scores, respectively. These may not fully reflect the absence of cognitive and physical impairment, however, their availability across all included cohorts permits pooling of harmonized subgroups.

In conclusion, we found in this IPDMA of population-based observational studies that a higher SBP is associated with a lower all-cause mortality in most octogenarian populations. Variation in the very old, embodied by cognitive and physical fitness, rather than cardiovascular status, changes the association between high SBP and lower all-cause mortality, however, SBP was not consistently associated with cognitive and functional decline. Since management of high BP should not be adapted solely on the basis of age alone, further research should focus on the benefits of targeting a higher SBP in older adults who are cognitively and physically less fit.

## ACKNOWLEDGEMENTS

### Substantial contributors

The LiLACS NZ study wishes to acknowledge the study participants, their families and whanau for supporting the study. We are extremely grateful Te RōpuKaitiaki o nga tikanga Maori for their guidance and we acknowledge the community partners (Western Bay of Plenty Primary Health Organisation, Nga Matapuna Oranga Kaupapa Maori Primary Health Organisation, Te Korowai Aroha Trust, Te Rūnanga o Ngati Pikiao, Rotorua Area Primary Health Services, Ngati Awa Research & Archives Trust, Te Rūnanga o Ngati Irapuaia and Te Whanau a Apanui Community Health Centre).

### Access to data statement

Request for access to the TULIPS consortium data are to be addressed to the corresponding author.

### Funding/support

The Leiden 85-plus study was partly funded by an unrestricted grant from the Dutch Ministry of Health, Welfare and Sports (1997–2001). The Life and Living in Advanced Age: a cohort study in New Zealand, Te Puawaitanga o Nga Tapuwae Kia Ora Tonu, was funded by the Health Research Council of New Zealand program grant (HRC 09/068B), Ministry of Health New Zealand (MOH ref: 345426/00), and Nga Pae o te Maramatanga (the New Zealand National Centre for Research Excellence for Maori; funded Maori engagement and project management) project grant, National Heart Foundation project grant for investigating cardiac markers and Oakley Mental Health Foundation project grant for investigating dementia. The Newcastle 85+ Study has been funded by the Medical Research Council, Biotechnology and Biological Sciences Research Council, the Dunhill Medical Trust and the National Institute for Health Research School for Primary Care. Parts of the work have also been funded by the British Heart Foundation, Unilever Corporate Research, Newcastle University, NHS North of Tyne (Newcastle Primary Care Trust). The Tokyo Oldest Old Survey on Total Health study was funded by, the Grant-in-Aid for Scientific Research (C) (MEXT KAKENHI, 21590775, 15KT0091), and Keio Global Research Institute (KGRI).

### Explanation of the role of funder/sponsor

The sponsors had no role in the design and conduct of the study, in the collection, analysis and interpretation of the data, nor in the preparation, review or approval of the manuscript.

### Conflicts of interest

There are no conflicts of interest.

## Supplementary Material

Supplemental Digital Content

## Supplementary Material

Supplemental Digital Content

## Supplementary Material

Supplemental Digital Content

## Supplementary Material

Supplemental Digital Content
