# Supplementary material for: Disentangling the varying associations between systolic blood pressure and health outcomes in the very old: an individual patient data meta-analysis
Source: J Hypertens. 2022 Jul 11;40(9):1786–94. doi: 10.1097/HJH.0000000000003219 (PMC9451840; doi:10.1097/HJH.0000000000003219)
Supplement: Supplemental Digital Content [file jhype-40-1786-s001.pdf]

Supplementary Table 1. Details on variables used in the analysis

Variable	Description
**Diabetes Mellitus (DM)**	We defined DM (yes/no) in all four studies as a self- and/or general practitioner reported history of DM, the use of antidiabetic medication or an abnormal blood glucose level at baseline.
**Blood Pressure (BP)**	In the Leiden 85-plus Study two manual BP measurements, while in a seated position, were made with a time range between measurements of approximately two weeks. In the LiLACS NZ study, BP was automatically measured three times on one occasion, in the supine position. In the Newcastle 85+ Study, BP was automatically measured three times during one physical assessment, while in a seated position. In TOOTH, BP was automatically measured two times during one medical examination, while in a seated position.
**History of Cardiovascular Diseases (CVD)**	In the Leiden 85-plus Study, the history of CVD was available from physician-reported history and electrocardiogram (ECG) data. In the LiLACS NZ study, the CVD data was obtained from a self-reported history, screening of general practitioner (GP) and hospitalization records, and ECGs.[1] In the Newcastle 85+ Study, GP records were screened and ECGs were made. In TOOTH, a history of CVD was obtained from a personal interview and the medical reports brought to that interview.
**Grip Strength (GS)**	A Jamar hand dynamometer (Sammons Preston INC., Illinois, USA) was used in the Leiden 85-plus Study. A Takei hand dynamometer Grip-D (Takei Scientific Instruments Co., Niigata-City, Japan) was used in the LiLACS NZ and Newcastle 85+ study. In TOOTH, a Tanita 6103 handheld dynamometer (Tanita cooperation, Tokyo, Japan) was utilized. The average value of two and the highest value of three measurements of the dominant hand was used in TOOTH and the Leiden 85-plus Study, respectively. The LiLACS NZ study obtained the average value of three measurements of the strongest hand. In the Newcastle 85+ Study, the mean recording of four measurements alternating between dominant and non-dominant hand were used
**Daily functioning**	In the Leiden 85-plus Study, ADL was measured with the Groningen Activity Restriction Scale (GARS).[2] The GARS consists of 18 items scored between 1 (fully independent) and 4 (only with help), adding up to a maximal score of 72 (worst). The LiLACS NZ study utilized the Nottingham Extended Activities of Daily Living (NEADL) questionnaire.[3] The NEADL scores 22 items and ranges from 0 (worst) to 22 (optimal). In the Newcastle 85+ study, ADL was evaluated with a sum score based on 17 activities with total scores ranging from 0 (optimal) to 17 (worst). For TOOTH, the Lawton Instrumental Activities of Daily Living was utilized, with a score ranging from 0 (worst) to 5 (optimal) based on 5 items.[4]

References of Supplementary Table 1

1. Teh R, Doughty R, Connolly M, Broad J, Pillai A, Wilkinson T, et al. Agreement between self-reports and medical records of cardiovascular disease in octogenarians. J Clin Epidemiol 2013;66:1135-43.
2. Kempen GI, Miedema I, Ormel J, Molenaar W. The assessment of disability with the Groningen Activity Restriction Scale. Conceptual framework and psychometric properties. Soc Sci Med 1996;43:1601-10.
3. Essink-Bot ML, Krabbe PF, Bonsel GJ, Aaronson NK. An empirical comparison of four generic health status measures. The Nottingham Health Profile, the Medical Outcomes Study 36-item Short-Form Health Survey, the COOP/WONCA charts, and the EuroQol instrument. Med Care 1997;35:522-37.
4. Lawton MP, Brody EM. Assessment of older people: self-maintaining and instrumental activities of daily living. Gerontologist 1969;9:179-86.
