# Supplementary material for: Disentangling the varying associations between systolic blood pressure and health outcomes in the very old: an individual patient data meta-analysis
Source: J Hypertens. 2022 Jul 11;40(9):1786–94. doi: 10.1097/HJH.0000000000003219 (PMC9451840; doi:10.1097/HJH.0000000000003219)
Supplement: Supplemental Digital Content [file jhype-40-1786-s002.pdf]

Supplementary Table 2. Pooled stratified estimates of the change in Mini-Mental State Examination scores per increase of systolic blood pressure with 10 mmHg, with every six months since baseline.

Outcome MMSE		β	95% C.I.		*P* value of difference
**Non-stratified**					
Initial Model		**n/a:** $I^2 = 72\%$			
**Stratified by cardiovascular status**					
**BP-lowering drugs**					
	Yes	**n/a:** $I^2 = 82\%$			n/a
	No	**0.02**	-0.01	0.05	
**History of CVD**					
	Yes	**0.01***	-0.03	0.04	0.72
	No	**0.01**	-0.01	0.04	
**Stratified by cognitive and physical fitness**					
**Baseline MMSE**					
	Low	**n/a:** $I^2 = 65\%$			n/a
	High	**-0.01**	-0.03	0.01	
**Grip Strength**					
	Low	**n/a:** $I^2 = 71\%$			n/a
	High	**0.00**	-0.02	0.02	
**Body Mass Index**					
	Low	**n/a:** $I^2 = 67\%$			n/a
	High	**0.01**	-0.01	0.03	

Repeated measures linear mixed model estimation of the change in Mini-Mental State Examination (MMSE) scores per increase of systolic blood pressure with 10 mmHg, with every six months since baseline. Models were corrected for sex (all) and age (only the Māori and TOOTH cohort). Both initial and stratified analyses after pooling using random-effects models with inverse-variance weighting are presented, including subgroup differences per analysis.

C.I.: confidence interval. BP: blood pressure. CVD: cardiovascular diseases. MMSE: Mini-Mental State Examination. n/a: not available. *: I2 = 40-60%. When not shown or not otherwise labeled: I^2 <40%.
