# Supplementary material for: Disentangling the varying associations between systolic blood pressure and health outcomes in the very old: an individual patient data meta-analysis
Source: J Hypertens. 2022 Jul 11;40(9):1786–94. doi: 10.1097/HJH.0000000000003219 (PMC9451840; doi:10.1097/HJH.0000000000003219)
Supplement: Supplemental Digital Content [file jhype-40-1786-s004.pdf]

A

Study or Subgroup	Estimate MMSE	SE	Weight	Estimate MMSE IV, Random, 95% CI
Leiden 85-plus Study	-0.093	0.077	20.6%	-0.09 [-0.24, 0.06]
LiLACS-NZ - Māori	-0.038	0.12	9.7%	-0.04 [-0.27, 0.20]
LiLACS-NZ - non-Māori	0.11	0.072	22.9%	0.11 [-0.03, 0.25]
Newcastle 85+ Study	-0.086	0.073	22.4%	-0.09 [-0.23, 0.06]
TOOTH	-0.037	0.069	24.4%	-0.04 [-0.17, 0.10]
**Total (95% CI)**			**100.0%**	**-0.03 [-0.10, 0.05]**

Heterogeneity: Tau² = 0.00; Chi² = 5.04, df = 4 (P = 0.28); I² = 21%
Test for overall effect: Z = 0.66 (P = 0.51)

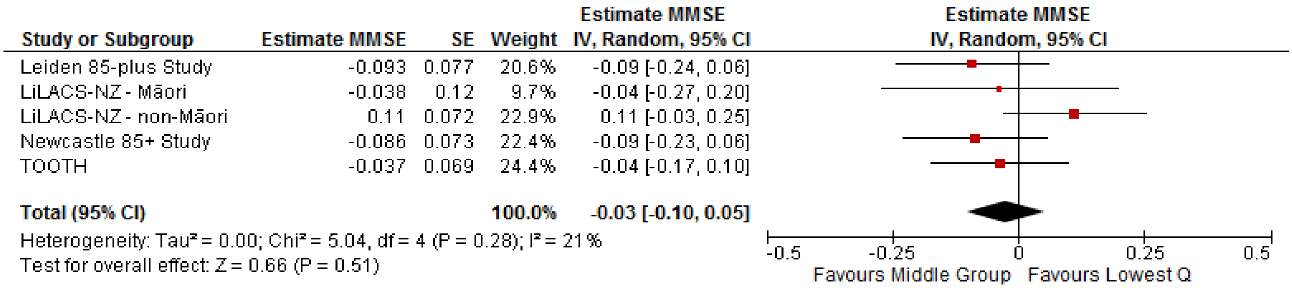

Study or Subgroup	Estimate MMSE	SE	Weight	Estimate MMSE IV, Random, 95% CI
Leiden 85-plus Study	0.133	0.073	20.8%	0.13 [-0.01, 0.28]
LiLACS-NZ - Māori	-0.186	0.106	14.5%	-0.19 [-0.39, 0.02]
LiLACS-NZ - non-Māori	0.042	0.074	20.6%	0.04 [-0.10, 0.19]
Newcastle 85+ Study	0.025	0.066	22.4%	0.03 [-0.10, 0.15]
TOOTH	-0.12	0.069	21.7%	-0.12 [-0.26, 0.02]
**Total (95% CI)**			**100.0%**	**-0.01 [-0.12, 0.09]**

Heterogeneity: Tau² = 0.01; Chi² = 9.88, df = 4 (P = 0.04); I² = 59%
Test for overall effect: Z = 0.21 (P = 0.83)

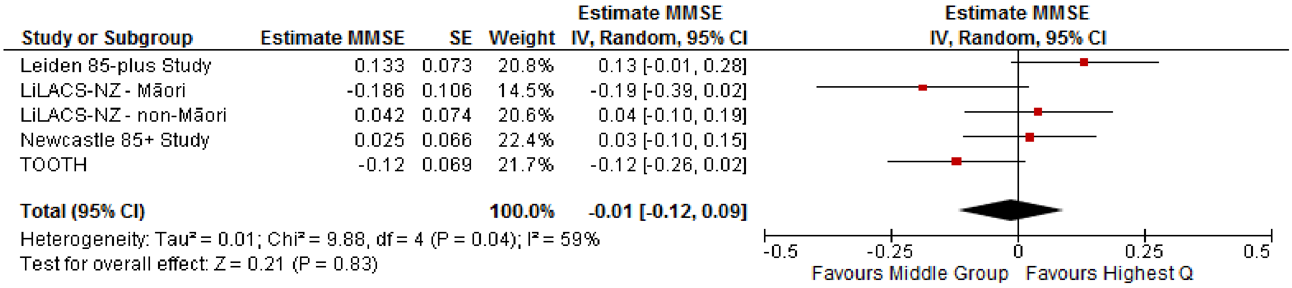

B

Study or Subgroup	Estimate ADL	SE	Weight	Estimate ADL IV, Random, 95% CI
Leiden 85-plus Study	-0.024	0.014	27.6%	-0.02 [-0.05, 0.00]
LiLACS-NZ - Māori	0.002	0.029	6.4%	0.00 [-0.05, 0.06]
LiLACS-NZ - non-Māori	-0.025	0.02	13.5%	-0.03 [-0.06, 0.01]
Newcastle 85+ Study	-0.022	0.012	37.5%	-0.02 [-0.05, 0.00]
TOOTH	-0.014	0.019	15.0%	-0.01 [-0.05, 0.02]
**Total (95% CI)**			**100.0%**	**-0.02 [-0.03, -0.01]**

Heterogeneity: Tau² = 0.00; Chi² = 0.85, df = 4 (P = 0.93); I² = 0%
Test for overall effect: Z = 2.75 (P = 0.006)

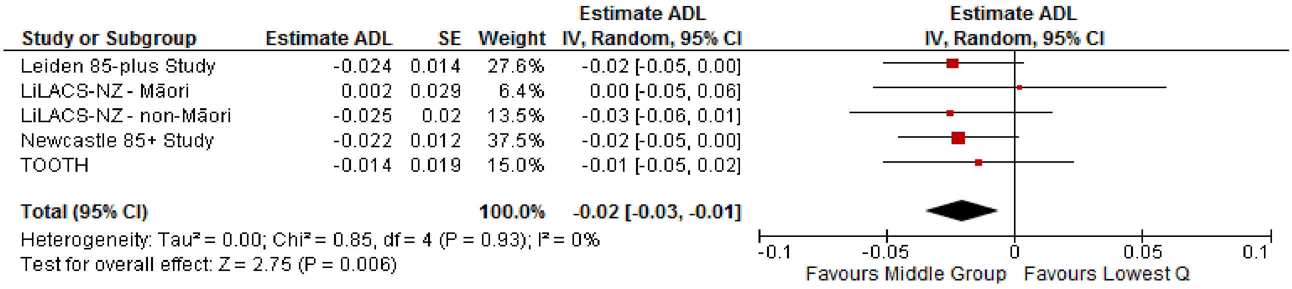

Study or Subgroup	Estimate ADL	SE	Weight	Estimate ADL IV, Random, 95% CI
Leiden 85-plus Study	0.024	0.013	26.6%	0.02 [-0.00, 0.05]
LiLACS-NZ - Māori	-0.036	0.026	10.6%	-0.04 [-0.09, 0.01]
LiLACS-NZ - non-Māori	-0.017	0.021	14.7%	-0.02 [-0.06, 0.02]
Newcastle 85+ Study	0.002	0.011	31.0%	0.00 [-0.02, 0.02]
TOOTH	-0.014	0.019	17.0%	-0.01 [-0.05, 0.02]
**Total (95% CI)**			**100.0%**	**-0.00 [-0.02, 0.02]**

Heterogeneity: Tau² = 0.00; Chi² = 6.52, df = 4 (P = 0.16); I² = 39%
Test for overall effect: Z = 0.18 (P = 0.86)

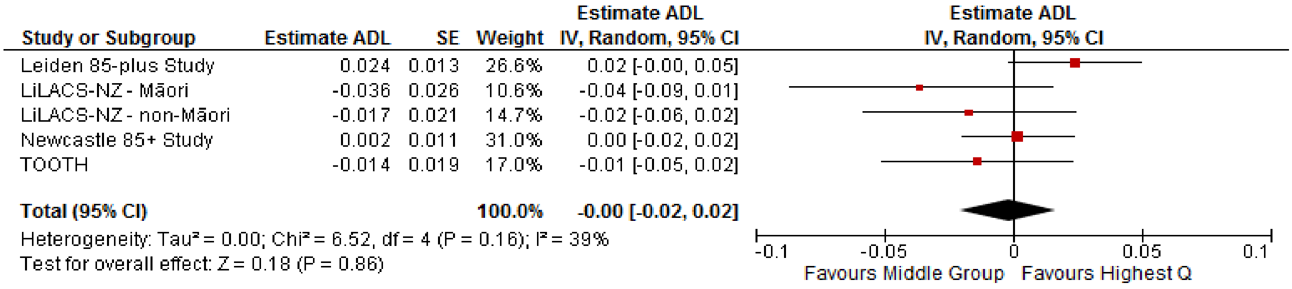

Supplementary Figure 1. Repeated measures linear mixed model estimation of the difference in (A) Mini-Mental State Examination (MMSE) scores and (B) standardized z-scores of activities of daily living (ADL) between the lowest and highest quartile (Q) versus the middle fifty percent group of systolic blood pressure with every six months since baseline. Models were corrected for sex (all) and age (only the Māori and TOOTH cohort). The estimates of the individual cohorts were pooled using a random-effects models with inverse-variance weighting.

SE: standard error. IV: inverse variance. CI: confidence interval.
